# Serum metabolomics profile identifies patients with community-acquired pneumonia infected by bacteria, fungi, and viruses

**DOI:** 10.1080/07853890.2024.2399320

**Published:** 2024-09-16

**Authors:** Li Chen, Jianbo Xue, Yukun He, Lili Zhao, Ying Zhang, Lu Yin, Shining Fu, Wenyi Yu, Xinqian Ma, Yu Wang, Yanfen Tang, Zhancheng Gao

**Affiliations:** a Department of Respiratory, Beijing Ditan Hospital, Capital Medical University, Beijing, People’s Republic of China; b Department of Respiratory & Critical Care Medicine, Peking University People’s Hospital, Beijing, China; c Department of Respiratory and Critical Care Medicine, Beijing Jishuitan Hospital, Beijing, China

**Keywords:** Metabolomics profiles, community-acquired pneumonia, bacterial infection, viral infection, fungal infection, diagnosis

## Abstract

**Purpose:**

Patients with bacterial, fungal, and viral community-acquired pneumonia (CAP) were studied to determine their metabolic profiles.

**Methods:**

Loop-mediated isothermal amplification technology and nucleic acid sequence-dependent amplification combined with microfluidic chip technology were applied to screen multiple pathogens from respiratory tract samples. Eighteen patients with single bacterial infection (B-CAP), fifteen with single virus infection (V-CAP), twenty with single fungal infection (F-CAP), and twenty controls were enrolled. UHPLC-MS/MS analysis of untargeted serum samples for metabolic profiles. Multiple linear regression and Spearman’s rank correlation analysis were used to determine associations between metabolites and clinical parameters. The sensitivity and specificity of the screened metabolites were also examined, along with their area under the curve.

**Results:**

The metabolic signatures of patients with CAP infected by bacteria, viruses, and fungi were markedly different from those of controls. The abundances of 45, 56, and 79 metabolites were significantly unbalanced. Among these differential metabolites, 11, 13, and 29 were unique to the B-CAP, V-CAP, and F-CAP groups, respectively. Bacterial infections were the only known causes of disturbances in the pentose and glucuronate and aldarate and ascorbate metabolism interconversions metabolic pathway.

**Conclusions:**

Serum metabolomic techniques based on UHPLC-MS/MS may identify differences between individuals with CAP who have been infected by various pathogens, and they can also build a metabolite signature for early detection of the origin of infection and prompt care.

## 1. Introduction

Community-acquired pneumonia (CAP), with an adult incidence of 30%–50%, remains the leading cause of infection-related death worldwide [1]. About 21% of patients require intensive care due to severe CAP [1, 2]. The clinical manifestations of CAP patients stratified by different severity levels vary widely [3]. Moreover, patient outcomes are highly heterogeneous due to differences in the infectious pathogens, as well as in the immune status of the host [4]. Therefore, disease diagnosis and treatment are difficult tasks. Unfortunately, technical limitations and hysteresis of pathogen testing as well as the emergence of multidrug-resistant bacteria may also create a huge waste of clinical medical resources, while increasing the probability of adverse drug reactions to antibiotics [5, 6]. Therefore, it is vital to explore the immune status of the patients infected by different types of pathogens as early as possible to help identify the pathogens of CAP infection, allowing for early and effective treatment and, ultimately, improved prognosis.

Metabolites in the organism are the most downstream products of different regulatory networks in the host, and their abundance changes ultimately reflect genomic and transcriptome regulatory networks, protein interaction networks as well as environment interactions [7]. Key roles in homeostasis and disease development are played by biological metabolites, which are engaged in processes such as host redox balance, oxidative stress, signal transduction, inflammation, and apoptosis [8]. Such metabolites may contribute to specific signatures in CAP [8–10]. Previous metabolomic studies have proven that metabolic dysregulation can be excavated in the peripheral blood of patients with CAP, and provided a comprehensive understanding of the pathophysiological mechanisms underlying pulmonary infectious diseases [11, 12]. However, there have been no studies conducted to date that look at how various infections affect the metabolic profiles of CAP patients. By elucidating this aspect, it may be possible to diagnose and treat patients more quickly and accurately, hence avoiding more serious consequences.

For this untargeted metabolomic study, we used high-performance liquid chromatography-mass spectrometry (HPLC-MS) to analyse serum samples from patients with CAP infected with different pathogens. A pathogen-related metabolic signature was identified, possibly paving the way for novel diagnostic and therapeutic strategies for CAP.

## 2. Materials and methods

### 2.1. Study design, demographics, and clinical information collection

Patients over the age of 18 who were hospitalized with CAP from January 2017 to October 2018, including at Peking University People’s Hospital (PKUPH), were included in this research. ClinicalTrials.gov has a record of this trial (NCT03093220). Participants were given information about the research and its potential risks and completed an written informed consent form in accordance with the Declaration of Helsinki. The PKUPH Institutional Review Board has evaluated and given their approval to this project (2016PHB202-01). We also established the criteria for enrolling patients with CAP by using the 2007 criteria released by the American/American Thoracic Society [13]. All cases enrolled for the research were CAP upon diagnosis and already hospitalized either at the respiratory or intensive care unit. CAP was defined by the following criteria: (1) a chest radiograph showing either a new patchy infiltrate, leaf or segment consolidation, ground glass opacity, or interstitial change; (2) at least one of the following signs – (a) the presence of cough, sputum production, and dyspnoea; (b) core body temperature >38.0 °C; (c) auscultatory findings of abnormal breath sounds and rales; or (d) peripheral white blood cell counts >10 × 10^9^/L or <4 × 10^9^/L; and (3) symptom onset that began in the community, rather than in a healthcare setting. Previous research served as the basis for the study’s exclusion criteria [11, 12]. The exclusion criteria were age <18 years, or the presence of any of the following: pregnancy, immunosuppressive condition, malignant tumour, end-stage renal or liver disease, active tuberculosis, or pulmonary interstitial fibrosis.

Lower respiratory tract specimens were collected during the first 24 h after hospital admission. Bronchoalveolar lavage (BAL) samples were obtained whenever possible within 7 days after admission. The presence of common respiratory pathogens were screened using an AgPath-ID^™^ One-Step real-time polymerase chain reaction (RT-PCR) kit (Ambion) with the FTD respiratory pathogens 21 kit (Fast Track Diagnosis, Luxembourg) [14]. A virus was considered as the etiology of CAP when the Ct value was < 30, using GAPDH as an internal control. Loop-mediated isothermal amplification (LAMP) assays were used to detect 13 common bacterial pathogens of CAP [14]. A bacterium was considered to be the causative pathogen only if the DNA concentration was over 10^4^ copies/mL. According to the results of pathogen detection, they were divided into a simple virus infection group, a simple bacterial infection group, a simple fungal infection group, and a mixed infection group. Only patients of the simple pathogen infection groups were included in this study.

The clinical baseline data of all subjects recruited were obtained from the medical record system, including basic disease information such as smoking history, age, gender and underlying diseases. White blood cell (WBC) count, lymphocyte percentage (LYM%), neutrophil percentage (NEU%), C-reactive protein (CRP), etc. data were also taken during the first 24 h of admission. In addition to the pneumonia severity index (PSI), the attending physician also recorded each patient’s disorientation, urea level, blood pressure, respiration rate, and age (CURB-65) scores to get a sense of the patient’s overall health and the extent of their disease.

### 2.2. Serum sample collection and preparation

Within the first 24 h of admission, blood from peripheral venous was pricked from patients with CAP, transported to the lab for fast separation of serum, and frozen at −80 °C until further investigation was possible. After that, the serum samples from all the patients were thawed on ice and vigorously vortexed to mix. To extract hydrophilic metabolites, 400 µL of pre-chilled methanol were mixed with 200 µL of a homogeneous serum sample. The procedure entails completely vortexing the mixture, storing it in a −80 °C freezer for incubation, removing it after 6-8 h, centrifuging it for 10 min (12,000 × g at 4 °C), and then lyophilizing the supernatant in vacuo for later analysis. At last, the sample was dried and placed at −80 °C until it was ready to be analyzed. Based on the detection of pathogens in patients’ respiratory tract samples, patients in the single virus infection (V-CAP), single bacterial infection (B-CAP), and single fungal infection group (F-CAP) were selected. The metabolite profiles of patient serum samples were analyzed in comparison with gender, sex-matched healthy control (HC) samples. After being dried out, the samples were reconstituted in 80 µL of 80% methanol (containing internal standards: Cholic acid-D4, Inosine-4N15, Meclofenamic acid, Stearic acid-D35, succinate-D4, Trp-D5, and uric acid), vortexed for 30 s, and then incubated at 4 °C for 15 min. Finally, the samples were centrifuged for 20 min at 12,000 × g at 4 °C, and the supernatant was collected for UHPLC-MS/MS analysis. In addition, 10 µL aliquots of each sample were combined and extracted in the same way as the primary samples to make mixed quality control (QC) samples. When testing the accuracy of the detection system, the QC samples were passed through the machine alongside the clinical samples.

### 2.3. Metabolite identification and untargeted UHPLC-MS analysis

The samples were analyzed using a UHPLC system Ultimate 3000 (Thermo Fisher, CA) and a Q Exactive (Orbitrap) mass spectrometer (Thermo Fisher, CA). Both positive mode (ESI+) and negative mode (DCI) were carried out using an ACQUITY UPLC BEH Amide column (1.7 μm, 2.1 × 100 mm, Waters) (ESI-). Additional file S1 provides information on metabolomics studies performed using LC-MS. Six quality control samples were utilized to calibrate the apparatus. Both positive and negative ion scanning modalities were used for detection.

Trace Finder 3.2 was used to apply the two tiers of raw data identification (Thermo Fisher Scientific, USA). Next, the raw data were processed while employing methods including baseline correction, peak signal recognition, denoising, spectral peak alignment, and segment integration. The exact mass of the obtained metabolites was compared to the mass spectrometry database kept in-house [15]. The in-house database contains ∼3500 endogenous metabolites which can be applied for MS identification. MS/MS standard spectra of ∼1500 metabolites were included for MS/MS confirmation. Two levels of metabolite identification were achieved based on the in-house database, one was MS/MS confirmation and the other was potential assignment based on accurate ion mass. It is true that the identification of metabolites mainly relied on the accurate ion masses and MS/MS matching with the database which was built from chemical standards. Only some of metabolites in the analysis were confirmed by retention times of chemical standards since most chemical standards are not sufficient for daily analysis and were used up when to establish the MS/MS library. In order to distinguish the confidence of metabolite assignment, the identifications were labeled as Level A-D [15]. Level A means confirmation using chemicals; Level B means potential assignment based on MS/MS; Level D means potential assignment according to accurate ion masses. In particular, only identified/possibly specified metabolites were used for statistical analysis. Ion features without annotation were not included in the analysis. Peak noise removal, baseline calibration, peak signal pickup, and peak area calculation were performed on the raw data (set the precursor mass tolerance <8ppm, matched fragment mass tolerance <15ppm). The retention time was allowed to shift by 0.25 min, and the identified metabolites were statistically analyzed to form a data matrix consisting of mass-charge ratio (m/z), retention time (RT), peak area and other information. Finally, the detected substance information was compared with the in-house database [15], and the library score was obtained to evaluate the reliability of the identification results. The closer the score is to 100, the better the match, the library score of less than 30 indicates low confidence in the results of metabolite identification.

### 2.4. Analysis and processing of untargeted UHPLC-MS/MS data

Data preprocessing was implemented to reduce interference information and mine meaningful biological information [16], and was performed on MetaboAnalyst 14.0 (https://www.metaboanalyst.ca) and SIMCA 4.0 software (Umetrics AB, Umea, Sweden). The main pretreatment steps are as follows: (1) Data screening: If a variable had a nonzero measurement value in at least 80% of the variables within one of the two subsets, the variable was included in the data set; otherwise the variable was removed. This procedure will be referred to as the ”80% rule”. Using this rule, missing values caused by peaks that were not present in the sample/chromatogram were reduced [17]. (2) Missing value filling: Since most of the missing value was caused by the low abundance of the compound (such as below the lower limit of detected abundance, the filtered data was filled as 1/2 of the detected minimum abundance value of the metabolite. (3) Calculate the coefficient of variation (CV): Variables with QC sample variation coefficient <30% were selected for subsequent analysis. (4) Data normalization: In order to reduce systematic bias in a given data set and improve the overall data consistency, we adopt the “by sum” method to normalize the test samples. (5) Data conversion and scaling: Log transformation was used to transform the data of compounds with large differences in detected values. Use Auto-scaling to ensure that all variables had the same impact during multivariate data analysis. After data preprocessing, the data showed a normal distribution for further analysis.

The data was put through a multivariate analysis (MVA) using SIMCA-P 14.0. A principal component analysis (PCA) model was built to demonstrate the approximate separation and aggregation of the data and to highlight outliers in order to evaluate the data’s credibility. A supervised orthogonal partial least squares discriminant analysis (OPLS-DA) model was built to further extract information that is unique to each group. The variable significance in projection (VIP) generated by the OPLS-DA algorithm indicated the relative contribution of each metabolite ion to the overall task of group classification. Our MVA model was put through a battery of 500 permutation tests and a cross-validation analysis of variance (CV-ANOVA) to ensure its accuracy. Differences in metabolite abundance between groups were tested for statistical significance using the Student’s t-test, and the false discovery rate (FDR adjusted p-values < 0.05) was calculated from the resulting p-values. Through OPLS-DA analysis, we determined the variable importance in projection (VIP) for each metabolite, which reflects the extent to which it helps differentiate the two groups. Highest potential metabolites for discriminating between the two groups were variables with VIP > 1 and FDR adjusted p-values < 0.05. Heatmaps depicting relative intensities of metabolites and pathway analysis of screened differential metabolites were generated using MetaboAnalyst 4.0 (http://www.metaboanalyst.ca/; Wishart Research Group, University of Alberta, Canada). The multi-collinearity in the data was corrected by a stepwise multiple linear regression (MLR) analysis, and a Spearman’s correlation heat map was created to investigate the relationship between metabolites and clinical infection markers.

### 2.5. Statistical analysis

Categorical variables are often expressed as percentages when used in statistical analysis. The Kolmogorov-Smirnov test confirmed the normal distribution of the continuous variables, which were then reported as mean ±standard deviation. Medians and interquartile ranges are used to characterise continuous data that do not follow a normal distribution (25th and 75th percentiles). The Chi-square test or Fisher’s exact test was used to determine statistically significant differences between the groups based on the categorical variables. Conversely, the Mann-Whitney U or Kruskal-Wallis tests were used to evaluate continuous variables across groups when they were not regularly distributed. Significant HPLC-MS/MS results were classified as having p-values less than 0.05 after using the Benjamini-Hochberg (BH) correction. MetaboAnalyst 4.0, SPSS statistics 19.0 (IBM, New York, USA), and MedCalc Software version 15.8 were used for all analyses (MedCalc Software, Ostend, Belgium).

## 3. Results

### 3.1. Participant demographics and clinical data

During the two-year study period, a total of 361 hospitalized patients diagnosed with CAP were collected, including 225 men (62.3%) and 136 women (37.7%), with a male-to-female ratio of 1.65: 1. The findings of the detection of the infectious pathogens show that the spectrum of infectious pathogens in CAP patients is complex and varied and that the variety of pathogen species is also a common feature of the CAP patient population. In the end, 20 patients of F-CAP, 15 cases of V-CAP, and 18 cases of B-CAP were chosen for further investigation (Additional file: Table S1). Twenty healthy people of same age and gender were also tested and found to be HC. Participants’ mean ages were 63.93 ± 8.51 years old. There was no statistically significant diff erence in gender, age, body mass index, smoking status, or preexisting illness across the groups. In addition, except for the clinical parameters of T_max_ and PCT, there was no significant statistical difference in the other laboratory examination indicators. Additional detailed demographic baseline characteristics and clinical information of all participants are presented in Table 1.

**Table 1.** The 73 participants’ demographic, clinical, and laboratory features.

Characteristic	**B-CAP** **(*N* = 18)**	**V-CAP** **(*N* = 15)**	**F-CAP** **(*N* = 20)**	**HC** **(*N* = 20)**	*p* value
Male sex—no. (%)	11 (61.10)	8 (53.33)	7 (35.00)	8 (40.00)	0.358^a^
Age—years	65.44 ± 13.93	60.60 ± 21.35	65.40 ± 17.36	60(48.25−67.00)	0.288^c^
BMI—(kg/m^2^)	22.50 ± 4.13	25.23 ± 11.54	23.47 ± 4.04	19.91 ± 2.50	0.492^b^
Smoking history—no. (%)	6 (33.30)	3 (15.00)	7 (46.70)	3 (15.00)	0.122^a^
**Underlying diseases—no. (%)**					
COPD	1 (5.60)	0	0	0	
Asthma	1 (5.60)	3 (15.00)	0	0	
Bronchiectasis	2 (11.10)	1 (5.00)	0	0	
Interstitial lung Disease	1 (5.60)	0	0	0	
Dyslipidemia	4 (22.20)	0	1 (6.70)	4 (20.00)	
Diabetes mellitus	5 (27.80)	4 (20.00)	5 (33.30)	2 (10.00)	
Hypertension	3 (16.70)	8 (40.00)	5 (33.30)	5 (25)	
Cardiovascular disease	3 (16.70)	3 (15.00)	0	2 (10.00)	
Cerebrovascular disease	1 (5.60)	0	2 (13.30)	0	
**Physical examination**					
T Max (°C)	37.94 ± 1.07	38.71 ± 1.31	37.99 ± 1.12	NA	0.045^b^
Respiratory frequency	20 (20−22)	20 (20−21)	20 (19−21)	NA	0.805^c^
Heart rate	89 ± 11	89 ± 14	87 ± 17	NA	0.914^b^
Lung rales—no. (%)	10 (58.80)	13 (65.00)	9 (60.00)	NA	0.919^a^
Disorder of consciousness—no. (%)	2 (11.80)	2 (10.00)	0	NA	0.407^a^
**Laboratory results**					
WBC (×10^9^/L)	11.17 ± 7.84	6.30 (5.10−6.89)	6.99 ± 3.56	NA	0.281^c^
NE (×10^9^/L)	9.14 ± 7.95	4.15 (2.93−4.95)	5.22 ± 3.68	NA	0.243^c^
LY (×10^9^/L)	1.26 ± 0.67	1.25 ± 0.50	1.15 ± 0.53	NA	0.793^b^
MO (×10^9^/L)	0.46 (0.26−0.65)	0.48 ± 0.33	0.41 ± 0.20	NA	0.564^c^
NEU (%)	73.35 ± 17.50	67.08 ± 13.97	69.25 ± 14.37	NA	0.465^b^
LYM (%)	18.02 ± 13.87	22.20 (14.65−28.30)	20.05 ± 11.00	NA	0.429^c^
MO percentages (%)	6.17 ± 2.95	7.57 ± 3.11	6.79 ± 2.72	NA	0.356^b^
NLR	4.10 (2−12.75)	3.00 (2.63−5.53)	3.10 (2.00−10.60)	NA	0.823^c^
PLR	153.80 (125.45−407.40)	138.25 (113.47−228.63)	205.47 ± 107.66	NA	0.557^c^
PCT (µg/L)	0.19 (0−3.37)	0 (0−0.15)	0.32 (0.02−1.78)	NA	0.042^c^
CRP (mg/L)	102.03 ± 117.33	57.23 ± 49.86	118.57 ± 116.31	NA	0.425^b^
PLT (×10^9^/L)	262.07 ± 145.54	164.36 ± 65.21	216.89 ± 70.33	NA	0.816^b^
PaO_2_ (mmHg)	74.81 ± 15.40	32.50 (0−84.90)	66.5 (66.00−67.00)	NA	0.947^c^
FiO_2_ (%)	32.29 ± 14.91	24.00 ± 32.89	21 (10.50−27.00)	NA	0.106^c^
PaO_2_/FiO_2_	2.25 ± 0.71	2.88 ± 1.25	3.20 ± 0.84	NA	0.764^b^
PaCO_2_(mmHg)	36.00 ± 9.84	0 (15.00−41.05)	34.30 ± 4.76	NA	0.274^c^
SaO_2_ (%)	95.30 ± 2.34	47.00 (0−97.08)	93 (46.00−98.45)	NA	0.703^c^
HCO_3_ (mmol/L)	24.34 ± 4.79	11.05 (0−24.43)	23.70 ± 7.13	NA	0.362^c^
**PSI**	80.44 ± 26.35	70.15 ± 26.53	73.40 ± 31.81	NA	0.523^b^
**CURB-65**	1 (1−2)	1 (0−1)	1(0−2)	NA	0.442^c^
**Hospitalization Days**	13.78 ± 7.42	8.00 (6.00−13.75)	15(10.00−16.00)	NA	0.054^c^
**30-day mortality-no. (%)**	3 (16.70)	0	1 (6.70)	NA	0.150^a^

**Abbreviations:** WBC: white blood cell; LY: lymphocyte; NE: neutrophil; NLR: neutrophil/lymphocyte ratio; MO: monocyte; PLT: blood platelet; PLR: platelet-lymphocyte ratio; PCT: procalcitonin; CRP: C-reactive protein; FiO_2_: Fraction of inspiration O_2_; PaO_2_: partial pressure of oxygen; SaO_2_: oxygen saturation; BMI: body mass index; COPD: chronic obstructive pulmonary disease; PSI: pneumonia severity index; CURB-65: confusion, urea, respiratory rate, blood pressure, and age ≥65 years old; NA: not applicable.

**Note:** Percentages are used to describe categorical variables. The mean ± standard deviation of normally distributed continuous variables was used in the Kolmogorov-Smirnov test. Medians and interquartile ranges are used to represent continuous variables that are not regularly distributed (25th and 75th percentiles).

^a^Fisher’s exact test or ^a^ Chi-square test; ^b^Analysis of variance using post-hoc Tukey HSD test, Student’s *t* test, Mann-Whitney *U* test, Kruskal–Wallis H test.

### 3.2. Serum metabolism profile of CAP patients from different groups

Untargeted LC-MS/MS analysis was used to examine the metabolic profiles of serum samples from 73 individuals distributed across four groups. Mass spectrometry was able to identify 604 different metabolites, including 360 metabolites in the ESI + mode and 244 metabolites in the ESI mode. Univariate analysis was performed on the obtained mass data of patient and HC samples. The serum of CAP patients was shown to have statistically significant variations in a large number of metabolites compared to the HC group. In the B-CAP, V-CAP, and F-CAP groups, the levels of 45, 56, and 79 metabolites were significantly dysregulated when compared with the HC group (FDR adjusted p-value <0.05).

### 3.3. Multivariate models established by untargeted metabolomics analysis

**Figure 1.**
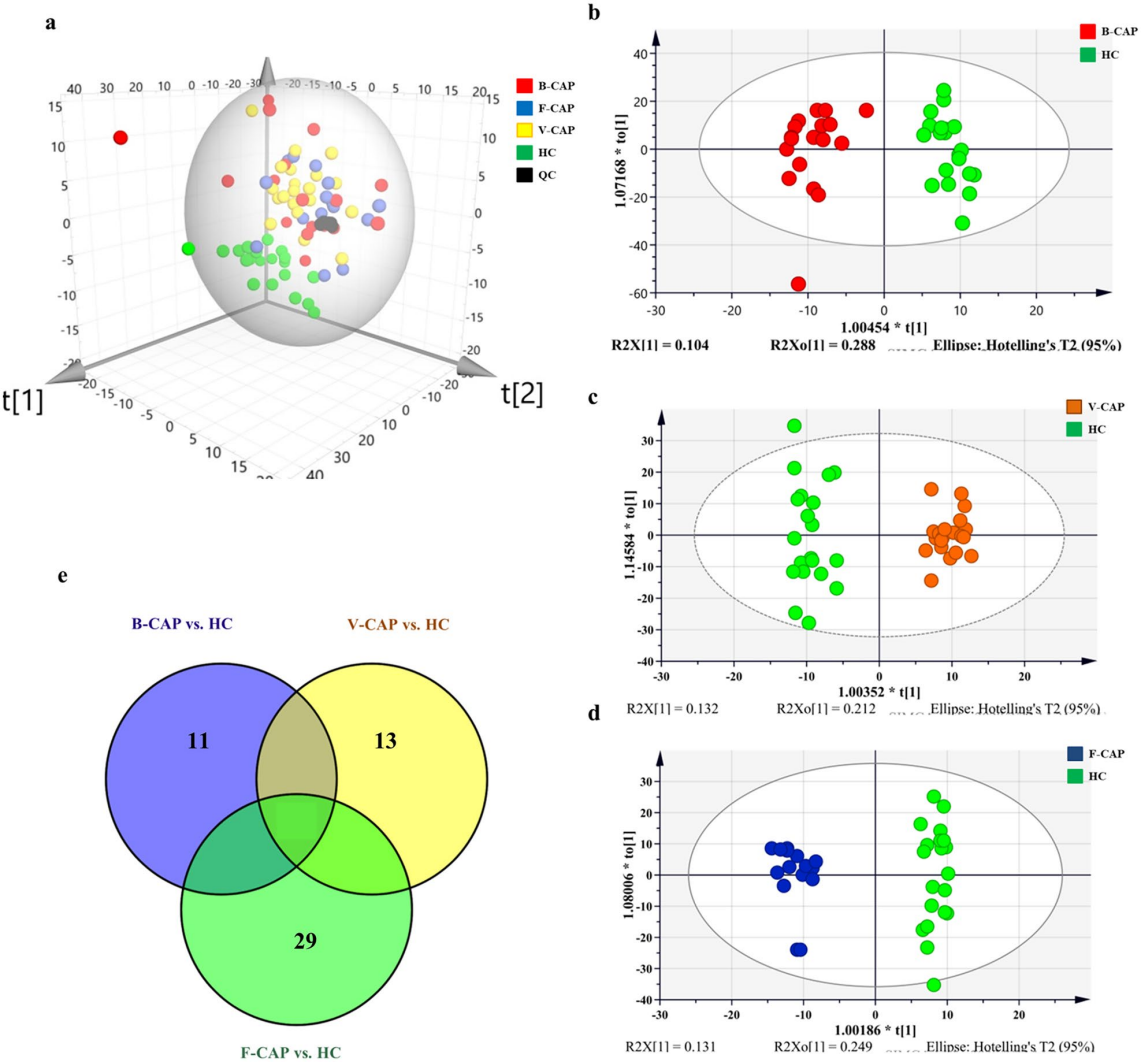
PCA scores plot and orthogonal partial least squares discriminant analysis (OPLS-DA) of metabolic profiles in CAP group (including B-CAP, V-CAP, F-CAP) and HC. The Venn diagram of the relationship between the data sets of differential metabolites in the three groups of patients with CAP compared to the HC group (a) PCA scores plot of metabolic profiles in CAP group (including B-CAP, V-CAP, F-CAP) and HC. (b) OPLS-DA score plot discriminates B-CAP versus HC (c) OPLS-DA score plots of V-CAP versus HC group. (d) OPLS-DA score plots of F-CAP versus HC. The model of OPLS-DA reflect good separation trends among CAP and HC. B-CAP, patients with single bacterial infection; V-CAP, patients with single virus infection; F-CAP, patients with single fungal infection. Red, B-CAP; orange, V-CAP; blue, F-CAP; green, healthy controls (HC). No sample was placed outside the ellipse that describes the 95% CI of hotelling’s T-squared distribution. (e) The Venn diagram visualizes the relationship between the data sets of differential metabolites in the three groups of patients with CAP compared to the HC group.

Multivariate statistical analysis (MVA) of the principal component analysis (PCA) (an unsupervised clustering approach) was performed to evaluate the general patterns in their distribution before any supervised statistical analysis was used. Analytical reproducibility and stability of the equipment in this UHPLC-MS study were confirmed by the tight clustering of quality controls from blood samples on the metabolomic profiles (Figure 1a). However, it is worth noting that the patient samples of all groups were diffusely distributed, and there was no obvious intra-group aggregation and inter-group segregation, which could be due to the high CAP-related heterogeneity in the metabolite profiles (Figure 1a). Furthermore, an OPLS-DA model was built to minimize the impact of confounding variables and maximize the extraction of differentiated metabolites. Good fitting and predicting performances were found using the OPLS-DA score plot (Figure 1b–d) to classify the various groups (Table 2). Notably, the metabolite profiles of patients with CAP varied significantly. Subsequently, permutation tests (500 iterations) were applied to the OPLS-DA model to assess its robustness and prevent data overfitting. The reliability of these models was evaluated using the parameters of the permutation tests. All OPLS-DA models had good predictive power (Q2 (sum)> 0.8) and strong explanatory ability (R2Y> 0.8) (Table 2 and Additional file: Figure S1). The p values obtained by CV-ANOVA were all <0.0001, reflecting obvious differences between the groups. The permutation test’s verification model had lower R2 and Q2 values than the original model did, and the Q2 regression line’s Y-intercept was greater than 0, demonstrating that the model was strong and free of overfitting. Hence, the constructed OPLS-DA models were reliable and could be used for subsequent statistical analysis of data and screening of differential metabolites.

**Table 2.** Evaluation parameters of our OPLS-DA models.

Group	Evaluation the constructed OPLS-DA models					Permutation test	
	PC^a^	R2X	R2Y	Q2(cum)	CV-ANOVA (*p*)	R2	Q2
CAP vs HC	1 + 4 + 0	0.476	0.979	0.685	<0.0001	0.913	−0.566
V-CAP vs HC	1 + 2 + 0	0.402	0.962	0.855	<0.0001	0.769	−0.507
F-CAP vs HC	1 + 2 + 0	0.440	0.976	0.870	<0.0001	0.765	−0.552
B-CAP vs HC	1 + 2 + 0	0.451	0.940	0.763	<0.0001	0.721	−0.560

a: PC The number of principal components in this model. PC represents the number of predicted components plus the number of orthogonal components.

Abbreviations: HC: healthy control; B-CAP: CAP of single bacterial infection; V-CAP: CAP of single virus infection; F-CAP: CAP of single fungal infection; CAP: community-acquired pneumonia.

**Figure 2.**
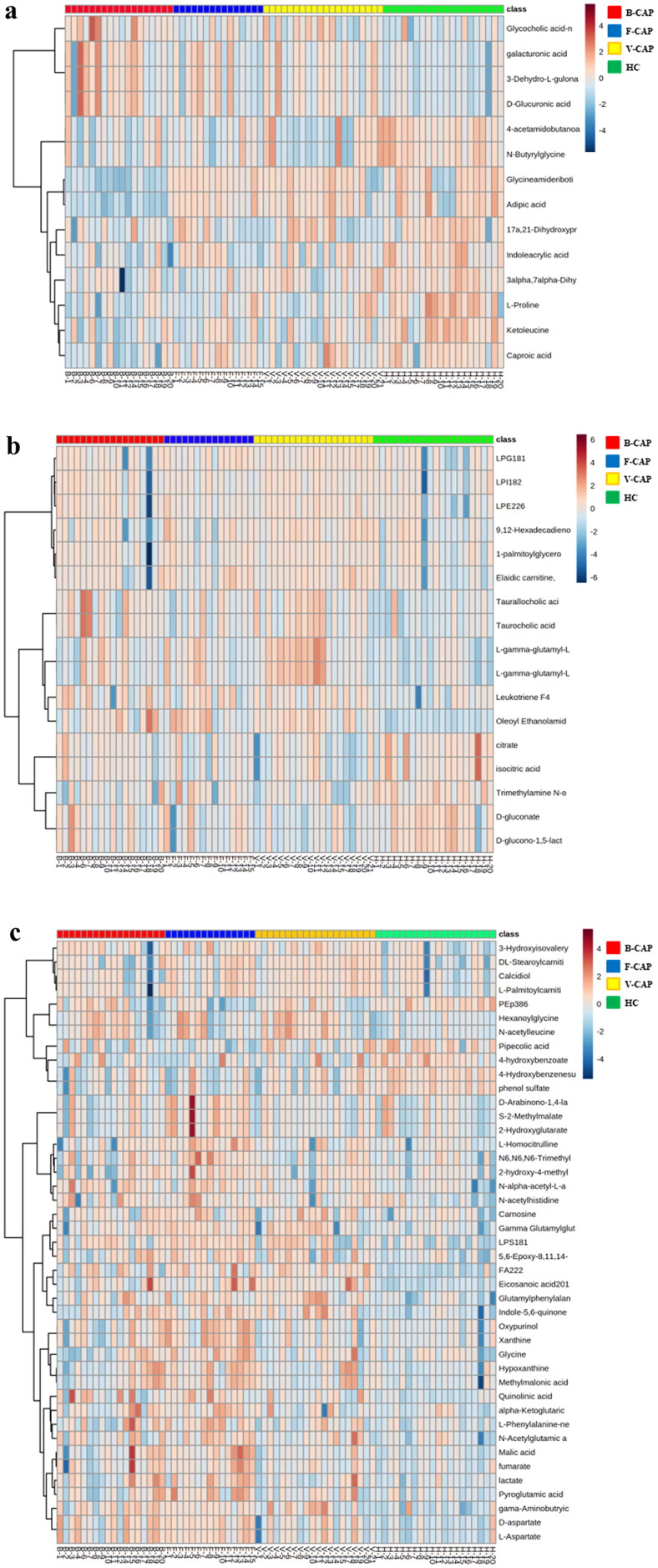
Hierarchical cluster heatmap of different metabolites unique to B-CAP (a), V-CAP (b), and F-CAP (c). Row represents metabolites and column represents individual samples. Red, blue, yellow and green represent B-CAP, V-CAP, F-CAP and HC, respectively. Greater brown indicates higher relative intensity of metabolites, while light blue indicates lower intensity.

According to the OPLS-DA model, we were able to determine the relative importance of each group. Patients with bacterial, viral, and fungal infections had their serum analyzed for differential metabolites using the cutoffs of VIP> 1 and FDR adjusted p-values < 0.05. When comparing the B-CAP, V-CAP, and F-CAP groups to HCs, a total of 45, 56, and 79 metabolites, respectively, were found to be substantially dysregulated (Additional file: Table S2). The robustness of the metabolomics analytical platform was shown by the wide range of CV values for these metabolites (3.20–29.80%), with a median of 7.70%. Metabolites showing statistically significant changes were then shown in a Venn diagram (Figure 1e). Surprisingly, the three groups of infected organisms showed remarkably varied levels of 11 metabolites, 29 metabolites, and 13 metabolites, respectively (Additional file: Tables S3–S5). On display in Figure 2 are heat-maps depicting the relative abundance of these distinct metabolites across the three treatment groups.

**Table 3.** Topological pathways of differential metabolites in the serum of patients with B-CAP.

Pathways	Total^a^	Hits^a^	Raw p value	Impact value
D-Glutamine and D-glutamate metabolism	6	2	0.008	1
Ubiquinone and other terpenoid-quinone biosynthesis	9	1	0.196	1
Ascorbate and aldarate metabolism	8	2	0.014	0.5
alpha-Linolenic acid metabolism	13	2	0.037	0.333
Alanine, aspartate and glutamate metabolism	28	1	0.495	0.197
Arginine and proline metabolism	38	4	0.011	0.187
Pentose and glucuronate interconversions	18	2	0.067	0.125
Tryptophan metabolism	41	3	0.072	0.118
Arginine biosynthesis	14	1	0.289	0.117

a. hits: The amount of metabolites enriched into this pathway; total: The total number of metabolites in this metabolic pathway.

### 3.4. Pathway enrichment analysis

**Figure 3.**
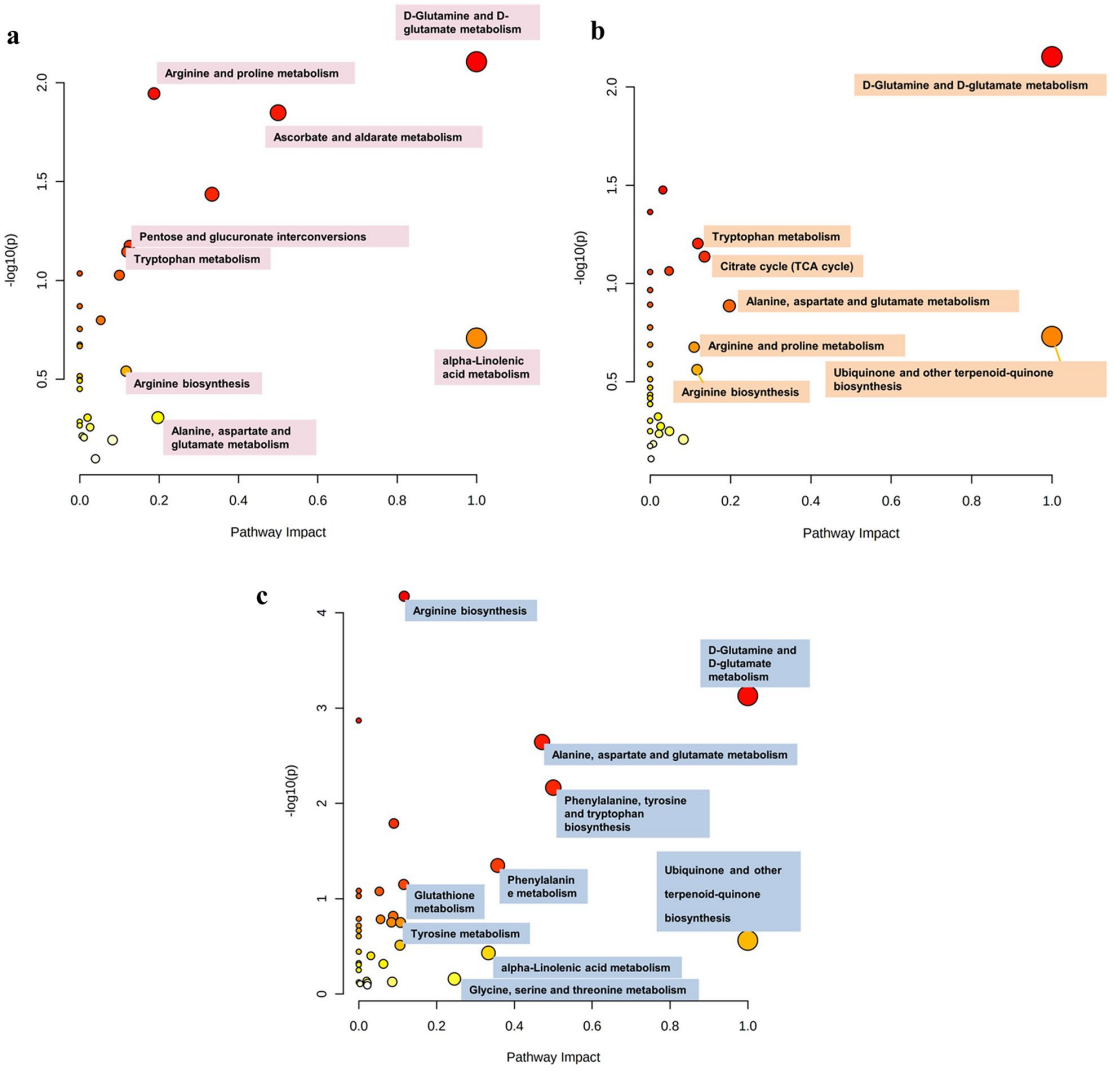
Metabolic pathway analysis and metabolic pathway network of different metabolites unique to three groups in CAP. Node colour based on p value and node radius determined based on pathway impact values. (a) Metabolic pathway analysis of different metabolites unique to B-CAP. (b) Metabolic pathway analysis of different metabolites unique to V-CAP. (c) Metabolic pathway analysis of different metabolites unique to F-CAP.

A topological analysis of metabolic pathways was performed based on the differential metabolites in each group. Pathway impact> 0.1 was defined as the critical pathway for infection by different types of pathogens. The enrichment pathways in each group are described in detail in Tables 3–5. Most metabolic alterations were in common between the three groups of patients, including changes in D-glutamine and D-glutamate metabolism, biosynthesis of ubiquinone and other terpenoid-quinones, as well as alanine, aspartate, and glutamate metabolism. It is worth noting that the unique differential metabolites in the different CAP groups also participate in the CAP metabolic pathway and constitute a unique metabolic pathway for this group of samples. For example, the metabolites galacturonic acid/glucuronic acid/3-Dehydro-L-gulonate, with unique alterations in the bacterial group, was involved in ascorbate and aldarate metabolism, as well as pentose and glucuronate inter-conversions. Similarly, the differential metabolites of the fungal and the viral infection groups also participate in many key pathways (Figure 3 and File S^2^).

**Table 4.** Lists the topological routes of several metabolites found in the serum of V-CAP patients.

Pathways	Total^a^	Hits^a^	Raw p value	Impact value
D-Glutamine and D-glutamate metabolism	6	2	0.0070217	1
Ubiquinone and other terpenoid-quinone biosynthesis	9	1	0.18624	1
Alanine, aspartate and glutamate metabolism	28	2	0.12997	0.197
Citrate cycle (TCA cycle)	20	2	0.073002	0.135
Tryptophan metabolism	41	3	0.062597	0.119
Arginine biosynthesis	14	1	0.27466	0.117
Arginine and proline metabolism	38	2	0.21083	0.110
Tyrosine metabolism	42	1	0.6218	0.083
Glycerophospholipid metabolism	36	1	0.56472	0.048
Pentose phosphate pathway	22	2	0.086314	0.047
Glyoxylate and dicarboxylate metabolism	32	3	0.033428	0.032
Glycine, serine and threonine metabolism	33	1	0.53312	0.026
Steroid hormone biosynthesis	85	2	0.58189	0.022
Glutathione metabolism	28	1	0.47545	0.020

a. hits: The amount of metabolites enriched into this pathway; total: The total number of metabolites in this metabolic pathway.

**Table 5.** Topological pathways of differential metabolites in the serum of patients with F-CAP.

Pathways	Total^a^	Hits^a^	Raw p value	Impact value
D-Glutamine and D-glutamate metabolism	6	3	0.001	1
Alanine, aspartate and glutamate metabolism	28	5	0.002	0.471
Phenylalanine, tyrosine and tryptophan biosynthesis	4	2	0.007	0.500
Glycine, serine and threonine metabolism	33	1	0.693	0.246
Phenylalanine metabolism	10	2	0.045	0.357
Glyoxylate and dicarboxylate metabolism	32	2	0.307	0.106
Arginine biosynthesis	14	5	<0.001	0.117
alpha-Linolenic acid metabolism	13	1	0.370	0.333
Glutathione metabolism	28	3	0.071	0.115
Tyrosine metabolism	42	3	0.178	0.107
Ubiquinone and other terpenoid-quinone biosynthesis	9	1	0.273	1

a. hits: The amount of metabolites enriched into this pathway; total: The total number of metabolites in this metabolic pathway.

### 3.5. Relationship between the level of differential metabolites and clinical parameters, and performance assessment

**Figure 4.**
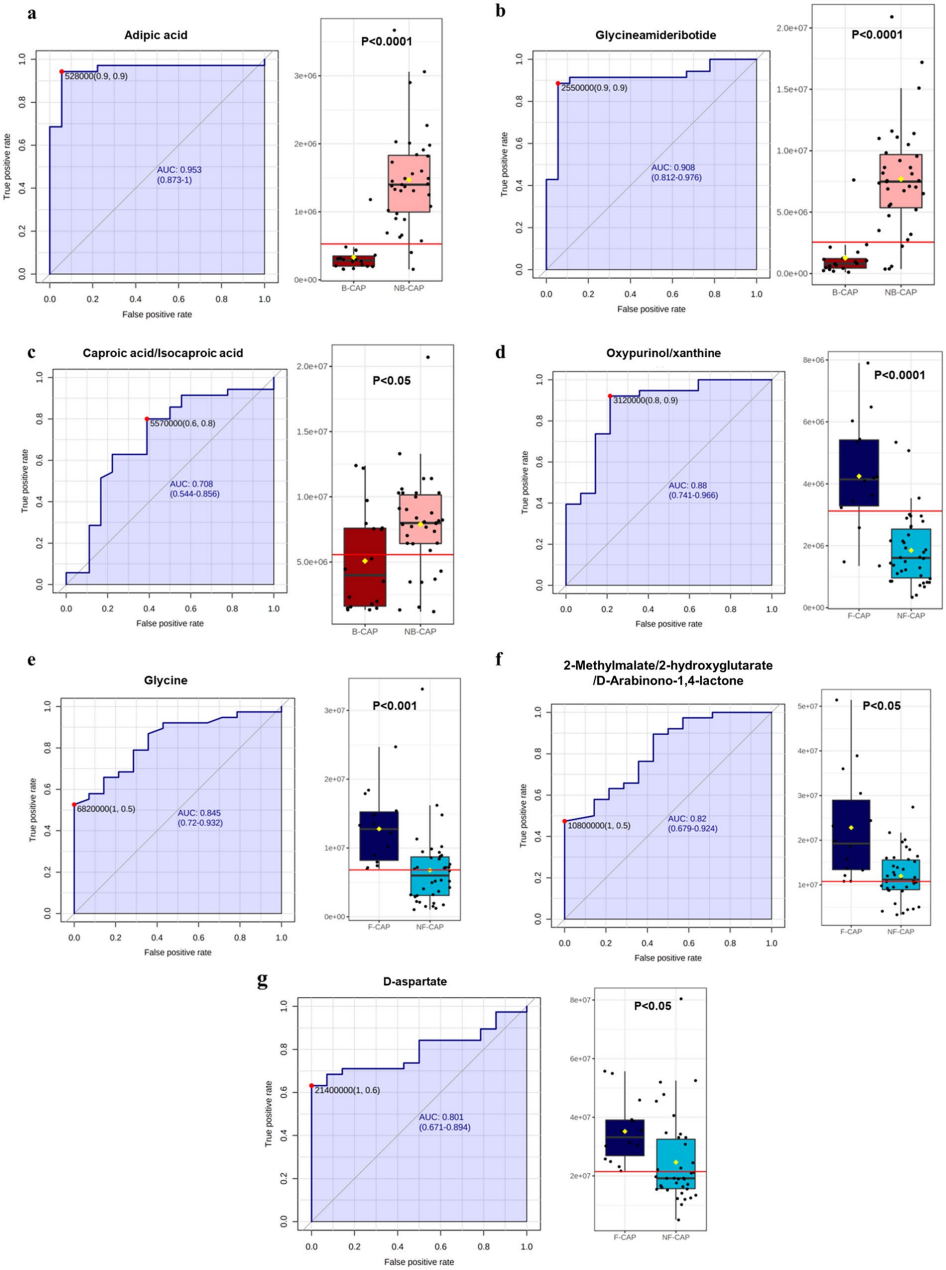
The area under the curve (AUC) of the ROC analysis and box plots of the selected different metabolites. (a-c) The area under the curves (AUCs) of the ROC analysis and box plot of the selected metabolites with excellent ability to distinguish B-CAP from other groups. (d-g) The AUCs and box plots of the selected metabolites with excellent ability to distinguish F-CAP from other groups.

ROC analysis was applied to verify whether the unique metabolite changes in the three groups could be effectively utilized to establish sensitive biological characteristics capable of distinguishing different pathogen types in CAP. Patients with CAP had considerably reduced levels of adipic acid and glycineamideribotide in their blood as compared to those without infection. When compared to PCT, CRP, WBC, and other clinical indications, the AUCs of adipic acid and glycineamideribotide were substantially higher, reaching as high as 0.935 (0.873-1) and 0.908 (0.812-0.976), respectively (Figure 4). However, numerous metabolites were more abundant in the fungal group than in the non-fungal group. For separating the fungus group from the non-fungal group, the AUC of oxypurinol/xanthine was as high as 0.880 (0.741-0.966). Figure 4 summarize the ability of the screened differential metabolites to predict CAP infection by different types of pathogens.

### 3.6. Unique metabolites and clinical parameters Correlation

**Figure 5.**
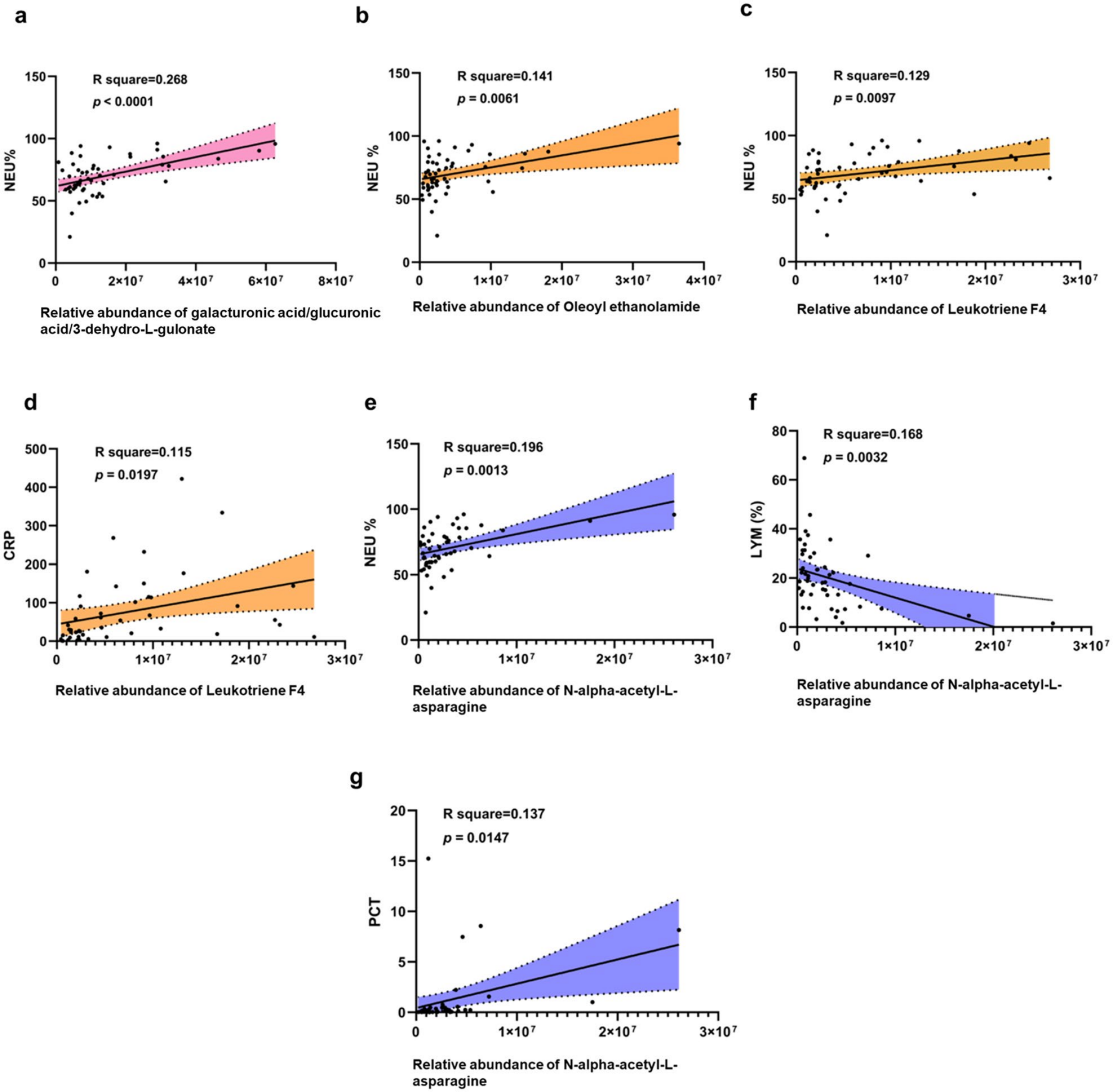
Correlation analysis between different metabolites unique to three groups and clinical parameters. (a) Correlation analysis of differential metabolites unique to B-CAP group and clinical parameters. (b-d) Correlation analysis of differential metabolites unique to V-CAP group and clinical parameters. (e-g) Correlation analysis of differential metabolites unique to F-CAP group and clinical parameters. Solid black line, the fitted regression line. Area within the dotted line lines, the 95% confidence intervals.

Spearman’s correlation analysis was used to examine the association between metabolites showing distinct changes across the three groups and the clinical parameters. The data showed that, in the bacterial infection CAP group, an increase in the relative levels of galacturonic acid/glucuronic acid/3-dehydro-L-gulonate, was positively related to NEU% (all adjusted *p* < 0.0001). Additionally, the relative abundance of the differential metabolites, oleoyl ethanolamide and leukotriene F4, screened in the viral infection group were positively correlated with NEU% (both *p* < 0.05). Moreover, the change in leukotriene F4 level exhibited a similar increase to that of CRP (*p* < 0.05). In patients with fungal infections CAP, N-alpha-acetyl-L-asparagine levels were considerably higher and showed positive correlations with NEU% and PCT but negative correlations with LYM and LYM% (Figure 5).

## 4. Discussion

Due to the diverse types of infectious pathogens that cause CAP, the host produces different degrees of immune response to the pathogens, making the disease outcome of patients with CAP highly heterogeneous. The development of metabolomics technology and its application in CAP diseases provide new ideas for studying the changes of metabolic characteristics in diseases, discovering characteristic biomarkers and exploring the pathophysiological changes of CAP. Studies have found that severe inflammatory processes can cause many changes in the metabolic level of cells [18]. The profiles of patients with sepsis 48h before death illustrated an obvious state of metabolic disorder, such that S-(3-methylbutanoyl)-dihydrolipoamide-E, phosphatidylglycerol (22:2 (13Z, 16Z)/0:0), glycerophosphocholine and S-succinyl glutathione were significantly decreased [19]. Ning P et al. demonstrated significant differences in metabolic characteristics between CAP patients and healthy controls by using UHLC tandem mass spectrometry. The abundance of 15 different metabolites was found to be significantly dysregulated, mainly involving the metabolic pathways of sphingolipid, arginine, pyruvate and inositol phosphate [11]. In addition, Zheng Y et al. performed metabolic profile and lipid profile analysis on alveolar lavage fluid collected within 72 h of admission from CAP patients. Pathway topological analysis showed that sphingolipid metabolic pathways were significantly disregulated in CAP patients, and the abundance of sphingomyelin and phosphatidylethanolamine was closely related to the proportion of phagocytes. Detection of metabolite abundance in host alveolar lavage fluid is helpful in differentiating patients with CAP and non-infectious lung disease [12]. However, there have been no studies on metabolomic differences in serum in patients with bacterial, fungal, or viral CAP infection. This research examined systemic metabolic alterations in CAP patients infected by different types of pathogens. To further understand how bacterial, fungal, and viral infections affect CAP patients’ metabolic profiles, we analyzed the serum of 73 people, including 53 CAP patients and 20 non-CAP controls. Serum may quickly, correctly, and intuitively indicate lung infection patients’ systemic metabolic alterations [12]. Untargeted metabolomics profiles in our study could clearly distinguished patients with CAP from age and sex matched HC, showing that the biochemical homeostasis of patients with CAP was significantly destroyed. The screening of small-molecule metabolites and investigation of the unique metabolic profile in the serum of CAP patients infected with various pathogens may provide light on the causes of disease variation. Moreover, this approach may provide ideas and identify therapeutic targets for the early screening and diagnosis of pneumonia pathogens, as well as for the development of new therapeutic options.

The combination of biomarker screening based on FDR adjusted p-values or p value and VIP calculation by using an OPLS-DA model as a standard has been widely used in previous metabolomics research [11]. The VIP values determined by the OPLS-DA model in our investigation reflected the individual contribution of each metabolite’s abundance to the observed group difference. The VIP > 1 and FDR adjusted p-values < 0.05 variables were judged to be novel metabolites. We used strictly limited criteria for screening and finally determined the different metabolites of CAP patients infected with different types of pathogens compared to the control group. A Venn diagram showing the overlapping differential metabolites of the three groups was constructed to better evaluate the shared richness. In our discovery cohort, the abundance of 45, 56, and 79 metabolites in bacteria, viruses, and fungi groups, respectively, changed significantly compared to the NC group. Of these variable metabolites, 11 substances were discovered to be exclusive to bacteria, while 13 and 29 were found to be viral and unique to fungi.

In the PCA, there was no evidence of either intra-group or inter-group variations in the serum metabolic profiles of CAP patients infected with various kinds of pathogens. Ascorbate and aldarate metabolism, as well as pentose and glucuronate interconversions, were shown to be possibly connected to the dysregulated metabolites in the bacterial infection group by pathway analysis. There were also notable changes in the metabolic pathways of citrate synthesis, glycerophospholipid metabolism, and other biochemical processes in individuals with viral infections. By transcriptional and metabolic profiling of human patients with sepsis, Cheng SC et al. found that a shift from oxidative phosphorylation to aerobic glycolysis was an important component of initial activation of host defense. In leukocytes rendered tolerant by exposure to lipopolysaccharide or after isolation from patients with sepsis and immunoparalysis, a generalized metabolic defect at the level of both glycolysis and oxidative metabolism was apparent, which was restored after recovery of the patients [20]. In this study, biosynthesis of phenylalanine, tyrosine, and tryptophan, as well as phenylalanine metabolism, all revealed distinct modifications in individuals with fungal infections. Regardless of the pathogenic pathogen, alterations in the metabolism of alanine, aspartate, and glutamate were seen in all CAP patient groups, indicating the activation of shared immune systems against pathogen infection and invasion. Importantly, it has been found that the products produced by the metabolic activities of the microbiota can provide signals to the host and may be involved in regulating the body’s immune response. Short-chain fatty acids, products of the gut microbiota, are recognized by the G-coupled protein receptor GPR43 expressed on innate immune cells and are involved in regulating the immune response in the lungs [21]. In addition, tryptophan, a metabolite produced by Lactobacillus Reuteri in the intestinal flora, can regulate intestinal mucosal immunity [22]. The tryptophan derivative indole 3-aldehyde acts as an aromatic receptor ligand, contributing to the expression of IL-22 and allowing Candida albicans to settle [23]. In addition, P. aeruginosa can produce metabolites that inhibit the formation of Aspergillus fumigatus biofilm [24]. The specific products contained in the sputum of patients with pulmonary cystic fibrosis are mainly composed of Pseudomonas aeruginosa metabolites and sphingolipids produced by the host [25]. Among them, host sphingolipids contain inflammatory mediators ceramide, which may play a potential sustained regulatory role in chronic inflammation.

The AUCs of the diagnostic effectiveness of these metabolites were determined using ROC, and the correlations between the differential metabolites and clinical data were investigated. Several of these metabolites have shown to be particularly useful in identifying infections caused by their related pathogens. Adipic acid was reported to be a metabolite of Escherichia, and its abundance changes significantly in diseases such as glutaric aciduria type 2 [26]. While adipic acid has been proposed as a possible biomarker of bacterial infection, to far there has been no experimental data supporting this claim. We found that adipic acid levels strongly and specifically predicted CAP-related bacterial infection, with an AUC of 0.953. (0.873-1). Similarly high predictive ability was shown by glycineamideribotide, a novel downregulated metabolite in the bacterial infection group (AUC = 0.908 [0.812-0.976]). Humans and microorganisms both have glycineamideribotide in their DNA [27]. Research on glycineamide ribotide in humans, however, is limited. Both PCT and CRP have been extensively studied for their use in the detection of bacterial infections [28]. Patients with hematologic malignancies who had bacterial infectious episodes had greater PCT and CRP levels than those who experienced non-bacterial events, according to research by Mina Yang et al. In the absence of neutropenia, both PCT and CRP demonstrated high diagnostic accuracy (AUC, 0.757 vs. 0.763, respectively) [28]. In this study, the predictive ability of adipic acid and glycineamideribotide for bacterial and non-bacterial pneumonia was far superior to clinical indicators such as WBC, NEU%, PCT, and CRP. At the same time, the abundance of LPE (22:6) and D-gluconate/D-glucono-1,5-lactone showed high predictive power when distinguishing the virus-infected group from the other groups, with an AUC of 0.670 (0.539–0.823) and 0.647 (0.480–0.787), respectively.

Correlation analysis revealed that the changes in the abundance of many differential metabolites were strongly correlated with clinical indicators. For example, changes in the concentrations of galacturonic acid/glucuronic acid/3-dehydro-L-gulonate, which was exclusive to the bacterial infection group, were positively correlated with NEU%. Galacturonic acid/glucuronic acid/3-dehydro-L-gulonate is widely present in bacteria and humans. Furthermore, galacturonic acid serves as both a food and a signal regulating the delicate microbiota-pathogen connections in the gut [29]. NEU% was positively associated with oleoylethanolamide concentration, which was shown to be considerably greater in the viral infection group compared to the non-infected group. Anti-inflammatory actions, immunological response, activation of lipolysis, and fatty acid oxidation are all linked to the bioactive lipid amide oleoylethanolamide, which is generated in the digestive tract [30, 31]. Epidemiological research on inflammatory illnesses suggests that oleoylethanolamide, an endocannabinoid-like lipid that interacts with peroxisome proliferator-activated receptor- and regulates anti-inflammatory activities, exists [32]. Samad Ghaffari found that giving patients exogenous oleoylethanolamide, a homeostatic signal that interferes with COVID-19 infection and reduces inflammation, improved their health [33]. Leukotriene F4 is a byproduct of the metabolism of arachidonic acid. In response to inflammation or infection, this lipid is synthesized by a series of events begun by cytosolic phospholipase A2, which frees arachidonic acid from phospholipids in the nuclear membrane [34]. Patients’ NEU% and CRP were strongly associated with their leukotriene F4 levels, which were considerably up-regulated in the virus group.

This study is just an initial step in the evaluation of pathogen types involved in CAP infection using a metabolomics approach. Even though our results are in good agreement with published research, the sample size used in this study was relatively small, and after screening the differential metabolites, it is necessary to further construct a validation cohort to verify its clinical significance. This study did not explore the specific role of different metabolite ratios in early identification of pathogen types, and further exploration is needed. Furthermore, the study only tested the serum metabolic profile at admission and, therefore, did not consider dynamic changes or response to treatment. Moreover, there is an urgent need to integrate multiple omics studies into CAP analysis to provide new ideas and strategies for the precise diagnosis and treatment of CAP.

Our findings provide further evidence that UHPLC-MS/MS-based metabolomics techniques may be utilized to effectively show metabolic alterations in patients with CAP infected by various pathogens. The found molecular metabolites may help in the development of precision medicine for CAP patients and in the search for other biomarkers of the disease.

## Supplementary Material

Supplemental Material

## Data Availability

The data underlying this study are available on figshare (https://doi.org/10.6084/m9.figshare.25287343.v1).
