# Supplementary material for: Serum metabolomics profile identifies patients with community-acquired pneumonia infected by bacteria, fungi, and viruses
Source: Ann Med. 2024 Sep 16;56(1):2399320. doi: 10.1080/07853890.2024.2399320 (PMC11407381; doi:10.1080/07853890.2024.2399320)

**Additional file S1**

**Supplemental Methods**

**Untra high-performance liquid chromatography-mass spectrometry (UHPLC-MS/MS) analysis**

After the dried samples were reconstituted, taken 40µl of the suspension from each sample and added it to a 2mL chromatographic injection bottle for on-machine testing.

All serum samples were analysed using an Ultimate 3000 UHPLC (Dionex) system coupled to a Thermo Q-Exactive (Orbitrap) mass spectrometer (Thermo Fisher Scientific, San Jose, CA, USA). The detection was performed in the positive and negative ion scanning modes, respectively. In positive mode (ESI+), ACQUITY UPLC BEH Amide column (1.7 μm, 2.1×100 mm, Waters) was used. Mobile phase A was prepared by dissolving 0.63 g of ammonium formate in 50 mL of HPLC-grade water, then adding 950 mL of high performance liquid chromatography (HPLC)-grade acetonitrile and 1 Μl of formic acid. Mobile phase B was prepared by dissolving 0.63 g of ammonium formate in 500 mL of HPLC-grade water, followed by 500 mL of HPLC-grade acetonitrile and 1 μL formic acid. The linear gradient was set as follows: 0 min, 1% B; 2 min, 1% B; 3.5 min, 20% B; 17 min, 80% B; 17.5 min, 99% B; 19 min, 99% B; 19.1 min, 1% B; 22 min, 1% B.

In negative mode (ESI-), BEH Amide column was also applied (1.7 μm, 2.1×100 mm, Waters). Mobile phase A was prepared by dissolving 0.77g of ammonium acetate in 50 mL of HPLC-grade water, then adding 950 mL of HPLC-grade acetonitrile. Adjust pH to 9.0 with ammonium hydroxide solution. Mobile B was prepared by dissolving 0.77 g of ammonium acetate in 500 mL HPLC-grade water, subsequently, adding 500 mL HPLC-grade acetonitrile and adjusting pH to 9.0 with ammonium hydroxide solution. And the linear gradient was as follows: 0 min, 5% B; 2 min 5% B; 4 min, 20% B; 18 min, 85% B; 19 min, 95% B; 21 min, 95% B; 21.1 min, 5% B; 25 min, 5% B.

The detailed mass spectrometer parameters are as follows: spray voltage, 3.5 KV for positive and 2.5 KV for negative mode; capillary temperature, 275°C for positive and 320°C for negative mode; sheath gas flow rate (arb), 35; aux gas flow rate (arb), 8; mass range (m/z), 70-1050 for positive and 80-1200 for negative mode; full MS resolution, 70, 000; MS/MS resolution, 17,500; top N, 10; NCE, 15/30/45; duty cycle, 1.2s.


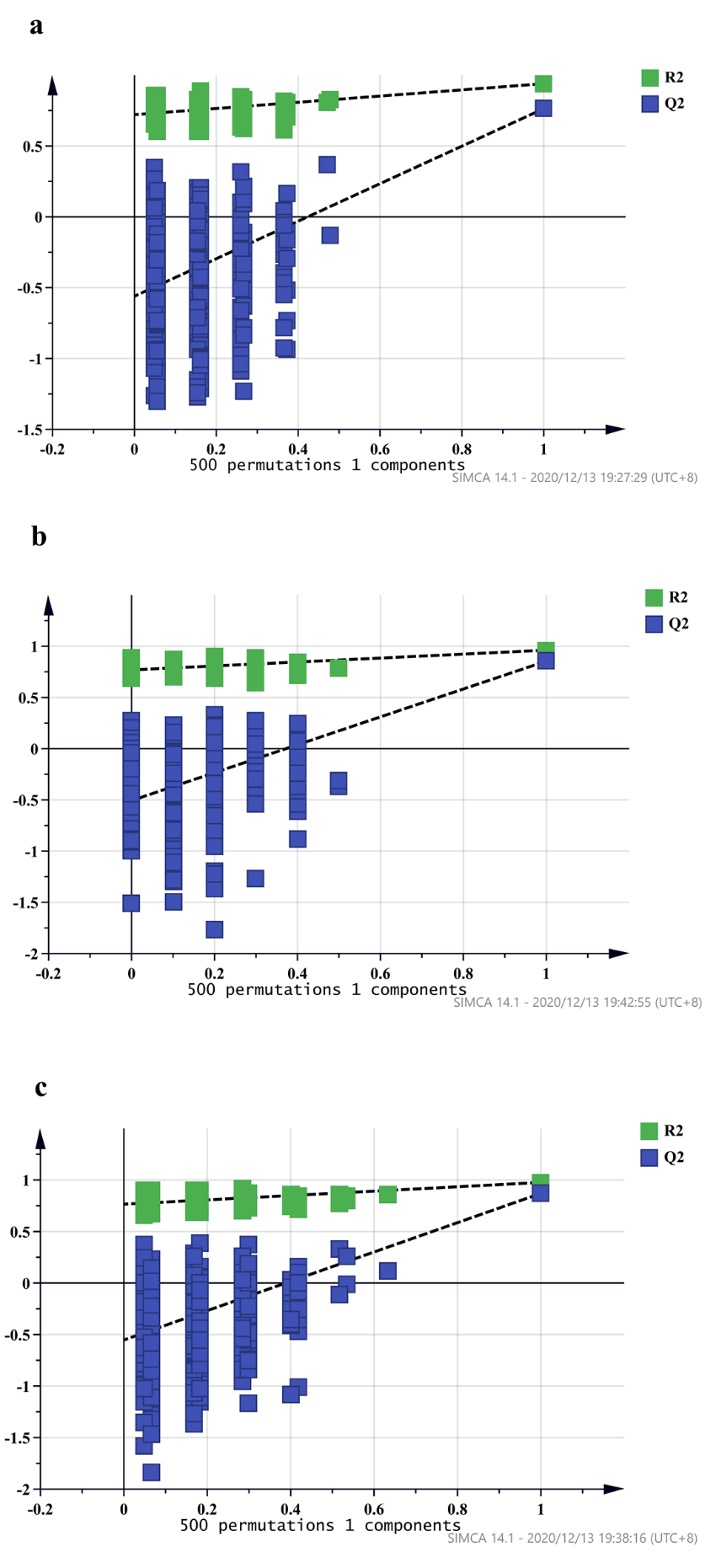

Supplement: Supplemental Material [file IANN_A_2399320_SM4057.zip › suppl_data/File S1.docx]
