# Supplementary material for: Serum metabolomics profile identifies patients with community-acquired pneumonia infected by bacteria, fungi, and viruses
Source: Ann Med. 2024 Sep 16;56(1):2399320. doi: 10.1080/07853890.2024.2399320 (PMC11407381; doi:10.1080/07853890.2024.2399320)
Supplement: Supplemental Material [file IANN_A_2399320_SM4057.zip › suppl_data/File S2.docx]

**Additional Table 1 Topological pathways of differential metabolites in the serum of patients with B-CAP**

| **Pathways** | **Total** | **Hits** | **Raw p value** | **Impact value** |
| --- | --- | --- | --- | --- |
| D-Glutamine and D-glutamate metabolism | 6 | 2 | 0.008 | 1 |
| Ubiquinone and other terpenoid-quinone biosynthesis | 9 | 1 | 0.196 | 1 |
| Ascorbate and aldarate metabolism | 8 | 2 | 0.014 | 0.5 |
| alpha-Linolenic acid metabolism | 13 | 2 | 0.037 | 0.333 |
| Alanine, aspartate and glutamate metabolism | 28 | 1 | 0.495 | 0.197 |
| Arginine and proline metabolism | 38 | 4 | 0.011 | 0.187 |
| Pentose and glucuronate interconversions | 18 | 2 | 0.067 | 0.125 |
| Tryptophan metabolism | 41 | 3 | 0.072 | 0.118 |
| Arginine biosynthesis | 14 | 1 | 0.289 | 0.117 |

**Additional Table 2 Topological pathways of differential metabolites in the serum of patients with V-CAP**

| **Pathways** | **Total** | **Hits** | **Raw p value** | **Impact value** |
| --- | --- | --- | --- | --- |
| D-Glutamine and D-glutamate metabolism | 6 | 2 | 0.0070217 | 1 |
| Ubiquinone and other terpenoid-quinone biosynthesis | 9 | 1 | 0.18624 | 1 |
| Alanine, aspartate and glutamate  metabolism | 28 | 2 | 0.12997 | 0.197 |
| Citrate cycle (TCA cycle) | 20 | 2 | 0.043002 | 0.135 |
| Tryptophan metabolism | 41 | 3 | 0.062597 | 0.119 |
| Arginine biosynthesis | 14 | 1 | 0.27466 | 0.117 |
| Arginine and proline metabolism | 38 | 2 | 0.21083 | 0.110 |
| Tyrosine metabolism | 42 | 1 | 0.6218 | 0.083 |
| Glycerophospholipid metabolism | 36 | 1 | 0.56472 | 0.048 |
| Pentose phosphate pathway | 22 | 2 | 0.086314 | 0.047 |
| Glyoxylate and dicarboxylate metabolism | 32 | 3 | 0.033428 | 0.032 |
| Glycine, serine and threonine metabolism | 33 | 1 | 0.53312 | 0.026 |
| Steroid hormone biosynthesis | 85 | 2 | 0.58189 | 0.022 |
| Glutathione metabolism | 28 | 1 | 0.47545 | 0.020 |

**Additional Table 3 Topological pathways of differential metabolites in the serum of patients with F-CAP**

| **Pathways** | **Total** | **Hits** | **Raw p value** | **Impact value** |
| --- | --- | --- | --- | --- |
| D-Glutamine and D-glutamate metabolism | 6 | 3 | 0.001 | 1 |
| Ubiquinone and other terpenoid-quinone biosynthesis | 9 | 1 | 0.273 | 1 |
| Phenylalanine, tyrosine and tryptophan biosynthesis | 4 | 2 | 0.007 | 0.500 |
| Alanine, aspartate and glutamate metabolism | 28 | 5 | 0.002 | 0.471 |
| Phenylalanine metabolism | 10 | 2 | 0.045 | 0.357 |
| alpha-Linolenic acid metabolism | 13 | 1 | 0.370 | 0.333 |
| Glycine, serine and threonine metabolism | 33 | 1 | 0.693 | 0.246 |
| Arginine biosynthesis | 14 | 5 | <0.001 | 0.117 |
| Glutathione metabolism | 28 | 3 | 0.071 | 0.115 |
| Tyrosine metabolism | 42 | 3 | 0.178 | 0.107 |
| Glyoxylate and dicarboxylate metabolism | 32 | 2 | 0.307 | 0.106 |


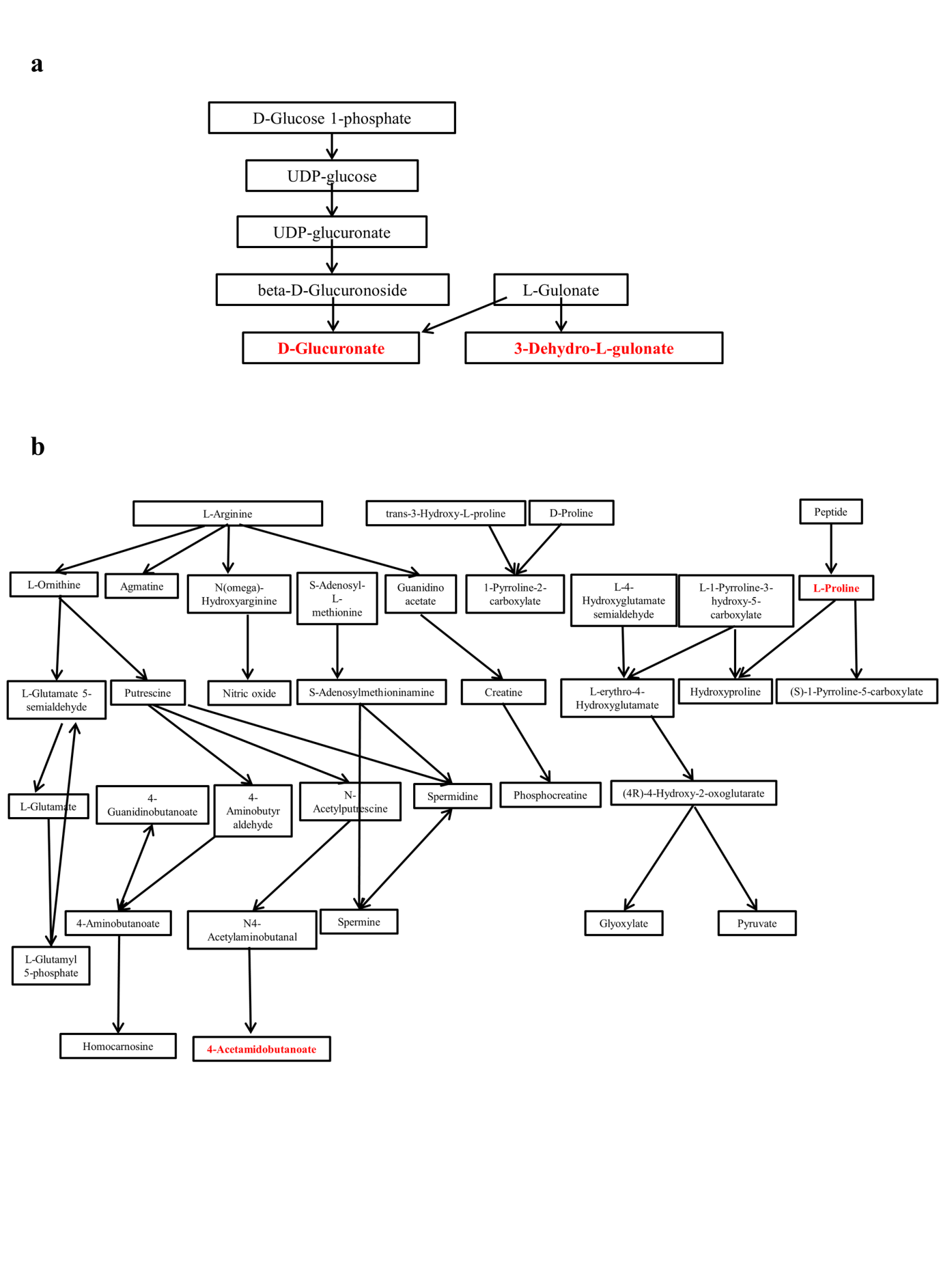


Fig. SS1 Topological pathway map of serum differential metabolites involved in B-CAP patients; Set screening conditions: P value <0.5, hits ≥2; Differential metabolites are marked red in the pathway map; a) D-Glutamine and D-glutamate metabolism; b) Arginine and proline metabolism;


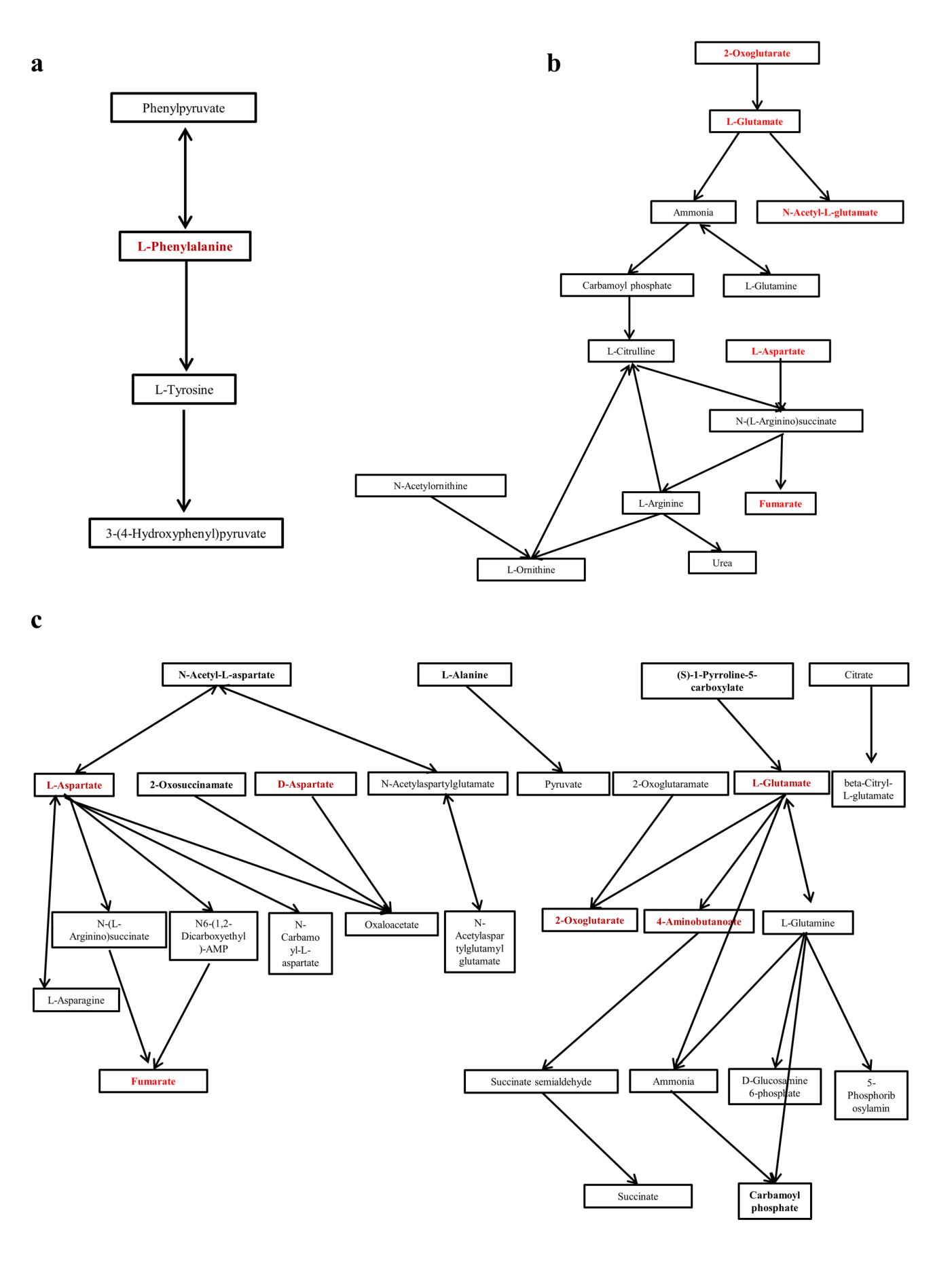


Fig. SS2 Topological pathway map of serum differential metabolites involved in F-CAP patients; Set screening conditions: P value <0.5, hits ≥2; Differential metabolites are marked red in the pathway; a) Phenylalanine, tyrosine and tryptophan biosynthesis; b) Arginine biosynthesis; c) Alanine, aspartate and glutamate metabolism;


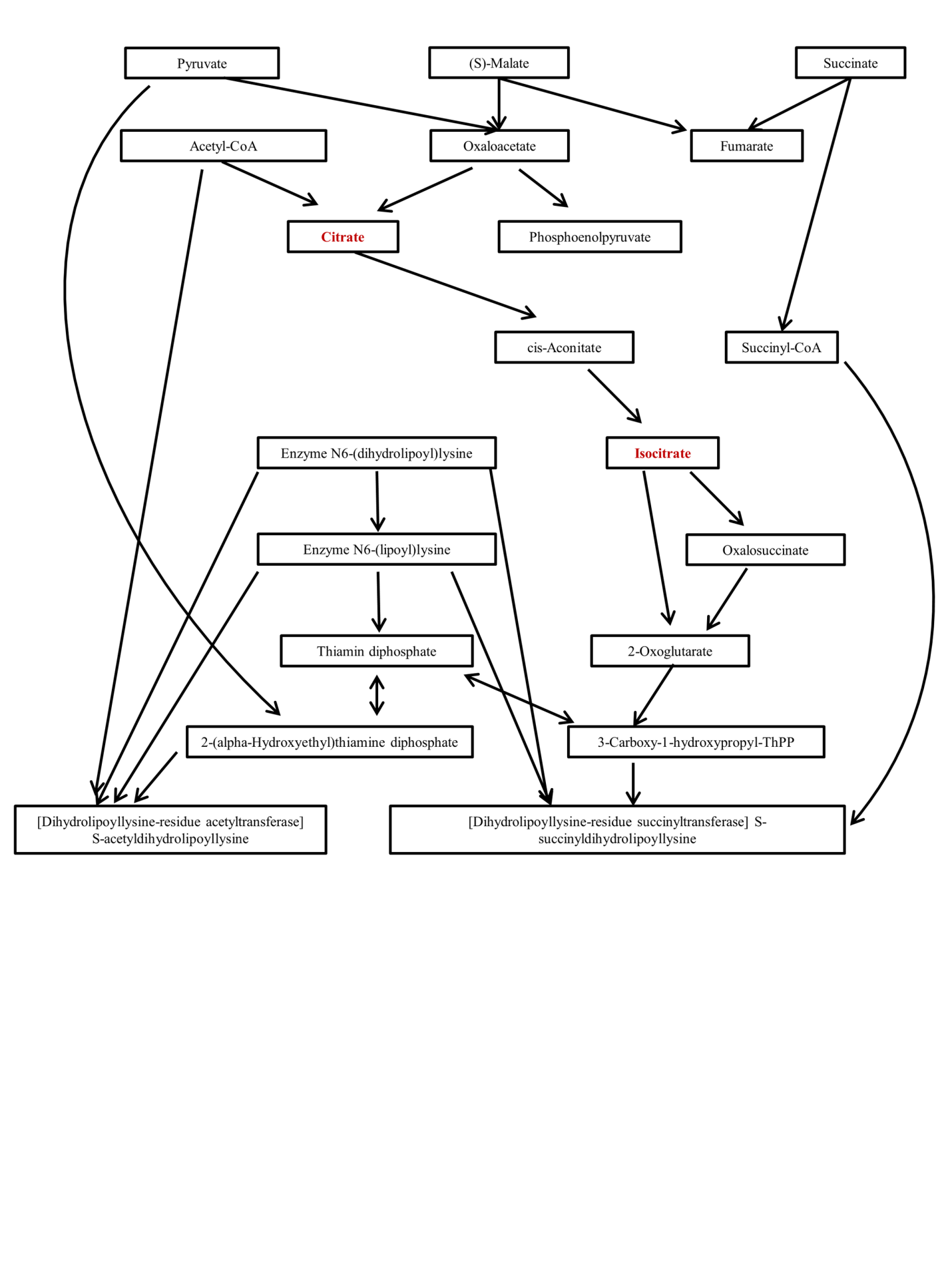


Fig. SS3 Topological pathway map of serum differential metabolites involved in V-CAP patients; Set screening conditions: P value <0.5, hits ≥2; Differential metabolites are marked red in the pathway; Citrate cycle (TCA cycle);
