# Supplementary material for: Serum metabolomics profile identifies patients with community-acquired pneumonia infected by bacteria, fungi, and viruses
Source: Ann Med. 2024 Sep 16;56(1):2399320. doi: 10.1080/07853890.2024.2399320 (PMC11407381; doi:10.1080/07853890.2024.2399320)
Supplement: Supplemental Material [file IANN_A_2399320_SM4057.zip › suppl_data/Table S3.docx]

Table S3 Unique differential metabolites in the serum of patients with bacterial infection of CAP

| **Compound** | **MODE** | **m/z (Expected)** | **RT** | **m/z (Delta (ppm))** | **VIP** | **FDR** | **B-CAP/HC Fold change** | **Match HMDB** |
| --- | --- | --- | --- | --- | --- | --- | --- | --- |
| Glycineamideribotide | neg | 285.04933 | 8.93 | 13.13703823 | 2.20563 | 0.001897 | 0.147827 | HMDB0002022 |
| Adipic acid | neg | 145.05063 | 8.91 | -1.491292911 | 1.90666 | 0.001206 | 0.207598 | HMDB0000448 |
| Indoleacrylic acid | neg | 186.05605 | 5.83 | -1.202210423 | 2.05039 | 0.00858 | 0.232874 | HMDB0000734 |
| 4-acetamidobutanoate/ N-Butyrylglycine | pos | 146.08169 | 2.28 | -7.626755949 | 1.72301 | 0.010144 | 0.304281 | HMDB0003681 |
| Ketoleucine | neg | 129.05517 | 4.23 | 3.24012481 | 1.12139 | 0.009755 | 0.546656 | HMDB0000695 |
| 3alpha,7alpha-Dihydroxy-5beta-cholestanate | pos | 435.34689 | 0.87 | -3.456027436 | 1.23292 | 0.02128 | 0.570055 | HMDB0000359 |
| 17a,21-Dihydroxypreg-nenolone | pos | 349.23734 | 0.89 | -0.889864079 | 1.60775 | 0.026905 | 0.572917 | HMDB0006762 |
| Caproic acid | neg | 253.2173 | 9.03 | -1.587995823 | 1.02132 | 0.047469 | 0.585439 | HMDB0000535 |
| L-Proline | pos | 116.07113 | 9.02 | -7.876789367 | 1.17118 | 0.039293 | 0.593805 | HMDB0000162 |
| galacturonic acid/glucuronic acid/3-dehydro-L-gulonate | neg | 193.03485 | 0.92 | 0.838058228 | 1.25708 | 0.047469 | 2.910806 | HMDB0002545 |
| Glycocholic acid | pos | 466.31631 | 7.09 | -3.676035714 | 1.81999 | 0.036263 | 8.740251 | HMDB0000138 |
