# Supplementary material for: Serum metabolomics profile identifies patients with community-acquired pneumonia infected by bacteria, fungi, and viruses
Source: Ann Med. 2024 Sep 16;56(1):2399320. doi: 10.1080/07853890.2024.2399320 (PMC11407381; doi:10.1080/07853890.2024.2399320)
Supplement: Supplemental Material [file IANN_A_2399320_SM4057.zip › suppl_data/Table S4.docx]

Table S4 Unique differential metabolites in the serum of patients with virus infection of CAP

| **Compound** | **MODE** | **m/z (Expected)** | **RT** | **m/z (Delta (ppm))** | **VIP** | **FDR** | **F-CAP/HC Fold change** | **Match HMDB** |
| --- | --- | --- | --- | --- | --- | --- | --- | --- |
| Trimethylamine N-oxide(TMAO) | pos | 76.07624 | 6.09 | -9.639421772 | 1.52997 | 0.024359 | 0.344559 | HMDB0000925 |
| Glycerophosphocholine | pos | 258.1101 | 11.65 | -3.982246076 | 1.03782 | 0.020962 | 0.399872 | HMDB0000086 |
| D-gluconate/D-glucono-1,5-lactone | neg | 177.03994 | 0.90 | 1.336335063 | 1.16894 | 0.010479 | 0.471858 | HMDB0000625 |
| Citrate/ isocitric acid | neg | 191.01921 | 0.90 | 1.552341646 | 1.04855 | 0.013881 | 0.609749 | HMDB0000094 |
| LPI (18:2) | neg | 595.28889 | 8.67 | -0.326738571 | 1.14957 | 0.04461 | 1.498494 | -- |
| LPG (18:1) | neg | 509.28873 | 9.17 | -2.215174521 | 1.41139 | 0.049526 | 1.682877 | -- |
| 9,12-Hexadecadienoylcarnitine | pos | 396.31138 | 4.67 | -5.380230633 | 1.31059 | 0.039451 | 1.79679 | HMDB0013334 |
| LPE (22:6) | neg | 524.27827 | 9.54 | -1.47567 | 1.40799 | 0.020646 | 1.821806 | HMDB0011496 |
| Leukotriene F4 | pos | 569.28911 | 3.47 | 1.736397792 | 1.35404 | 0.037373 | 2.216372 | HMDB0006465 |
| Elaidic carnitine,Faccenyl carnitine | pos | 426.35781 | 4.60 | -4.18023557 | 1.53673 | 0.030003 | 2.532968 | HMDB0006464 |
| L-gamma-glutamyl-L-isoleucine/  L-gamma-glutamyl-L-leucine | pos | 261.14505 | 9.60 | -5.938996203 | 1.32698 | 0.008248 | 2.585531 | HMDB11170 |
| taurallocholic acid/taurochloride | neg | 514.2844 | 7.15 | -2.2066045 | 1.56972 | 0.041649 | 3.453589 | HMDB0000922 |
| Oleoyl Ethanolamide | pos | 326.30535 | 0.85 | -4.116260506 | 2.02437 | 0.000372 | 4.235491 | HMDB0012088 |
