# Supplementary material for: Serum metabolomics profile identifies patients with community-acquired pneumonia infected by bacteria, fungi, and viruses
Source: Ann Med. 2024 Sep 16;56(1):2399320. doi: 10.1080/07853890.2024.2399320 (PMC11407381; doi:10.1080/07853890.2024.2399320)
Supplement: Supplemental Material [file IANN_A_2399320_SM4057.zip › suppl_data/Table S5.docx]

Table S5 Unique differential metabolites in the serum of patients with fungal infection of CAP

| **Compound** | **MODE** | **m/z (Expected)** | **RT** | **m/z (Delta (ppm))** | **VIP** | **FDR** | **F-CAP/HC Fold change** | **Match HMDB** |
| --- | --- | --- | --- | --- | --- | --- | --- | --- |
| Pipecolic acid | neg | 128.07118 | 1.48 | 2.775962911 | 1.16112 | 0.024159 | 0.487621 | HMDB0000070 |
| 4-Hydroxybenzenesulfonic acid/ phenol sulfate | neg | 172.9914 | 5.22 | -1.492616456 | 1.77492 | 0.006733 | 0.649019 | -- |
| PE (p38:6) | pos | 748.52812 | 5.180882 | -4.920002025 | 1.2896 | 0.046708 | 0.701579 | -- |
| FA (22:2) | neg | 335.2962 | 10.78 | -3.209374051 | 1.07688 | 0.011912 | 1.692136 | -- |
| L-Phenylalanine | neg | 166.08678 | 7.947592 | -7.342711519 | 1.02898 | 0.00194 | 1.745883 | HMDB0000159 |
| Pyroglutamic acid | neg | 128.03477 | 1.01 | 3.132917722 | 1.03197 | 0.006356 | 1.75735 | HMDB0000267 |
| fumarate | neg | 115.00316 | 0.90 | 3.004803418 | 1.04337 | 0.008517 | 1.76547 | HMDB0000134 |
| gama-Aminobutryic acid | neg | 104.07061 | 9.262776 | -3.287359241 | 1.10879 | 0.000475 | 1.843078 | -- |
| lactate | neg | 89.0239 | 0.97 | 5.045435696 | 1.06887 | 0.012037 | 1.858412 | HMDB0000190 |
| alpha-Ketoglutaric acid | neg | 145.01425 | 0.91 | -1.384080759 | 1.07379 | 0.047617 | 1.869239 | HMDB0000208 |
| N-Acetylglutamic acid | neg | 188.05645 | 0.91 | -0.974771266 | 1.21908 | 0.014291 | 1.968335 | HMDB0001138 |
| L-Aspartate | neg | 132.02971 | 0.92 | 2.71534481 | 1.38507 | 9.09E-05 | 2.133039 | HMDB0000191 |
| Glycine | pos | 76.03983 | 9.965407 | -8.846202658 | 1.07205 | 0.000494 | 2.145949 | HMDB0000123 |
| Malic acid | neg | 133.01373 | 0.90 | 2.68212557 | 1.29851 | 0.002604 | 2.294028 | HMDB0000744 |
| Glutamylphenylalanine | pos | 295.1294 | 9.704651 | 0.857132152 | 1.12289 | 0.00465 | 2.324254 | HMDB0029156 |
| Oxypurinol/ Xanthine | neg | 153.04071 | 5.625328 | -4.832714167 | 1.29817 | 0.004737 | 2.393453 | HMDB0000786 |
| (S)-2-Methylmalate/2-Hydroxyglutarate/  D-Arabinono-1,4-lactone | neg | 147.0299 | 0.90 | -1.561410633 | 1.08439 | 0.021442 | 2.439625 | METPA0309 |
| N6,N6,N6-Trimethyl-L-lysine | pos | 189.15975 | 12.84382 | -3.724635696 | 1.12362 | 0.000671 | 2.514252 | HMDB0001325 |
| DL-Stearoylcarnitine | pos | 428.37346 | 4.42191 | -2.186084054 | 1.2588 | 0.016392 | 2.573044 | HMDB0000848 |
| N-acetylhistidine | pos | 198.08787 | 9.795241 | -3.122598333 | 1.0381 | 0.006733 | 2.654024 | HMDB0032055 |
| L-Palmitoylcarnitine | pos | 400.34214 | 4.54695 | -4.003248182 | 1.09325 | 0.017679 | 2.65419 | HMDB0000222 |
| Hypoxanthine | pos | 137.04631 | 4.581008 | -7.73153481 | 1.27554 | 0.001891 | 2.772311 | HMDB0000157 |
| Calcidiol | pos | 401.34141 | 4.589277 | 4.441533544 | 1.17628 | 0.016392 | 2.794767 | HMDB0003550 |
| Quinolinic acid | neg | 166.01458 | 0.91 | -1.677313165 | 1.42143 | 0.000256 | 2.835272 | HMDB0000232 |
| 3-HydroxyisoFalerylcarnitine | pos | 262.16492 | 8.530131 | -4.043896795 | 1.13176 | 0.019614 | 2.843874 | HMDB0061189 |
| 4-hydroxybenzoate | neg | 137.0239 | 5.01 | 2.23066519 | 2.36508 | 0.013421 | 3.337853 | HMDB0000500 |
| Indole-5,6-quinone | pos | 148.0393 | 10.3228 | 7.04673137 | 1.63999 | 0.000354 | 3.413089 | HMDB0006779 |
| Methylmalonic acid | pos | 119.03389 | 4.58342 | 6.132224872 | 1.46745 | 0.000464 | 3.432272 | HMDB0000202 |
| Gamma Glutamylglutamic acid | neg | 275.08847 | 0.82 | -1.815131594 | 1.90189 | 8.09E-05 | 3.632561 | HMDB11737 |
